# Effects of Pelargonium Sidoides Extract on Apoptosis and Oxidative Stress in Human Neuroblastoma Cells

**DOI:** 10.3390/medicina60122110

**Published:** 2024-12-23

**Authors:** Ali Aslan, Mücahit Seçme

**Affiliations:** 1 Department of Physiology, Faculty of Medicine, Ordu University, Ordu 52200, Turkey; 2 Department of Medical Biology, Faculty of Medicine, Ordu University, Ordu 52200, Turkey; mucahitsecme@odu.edu.tr

**Keywords:** neuroblastoma, *P. sidoides*, cell line, SH-SY5Y, oxidative stress, apoptosis

## Abstract

*Background and Objectives:* Neuroblastoma is the most common extracranial solid tumor in children, often presenting challenges in treatment due to its clinical and genetic heterogeneity. This study investigated the anticancer potential of *Pelargonium sidoides* root extract on the human neuroblastoma cell line (SH-SY5Y). Using XTT assays, ELISA-based oxidative stress markers, and RT-PCR analysis of apoptotic genes, the study explored the extract’s effects on cell proliferation, oxidative stress, and apoptosis. *Materials and Methods:* For the cell culture, SH-SY5Y human neuroblastoma cells were thawed, cultured, and maintained under appropriate conditions for experiments. The dose- and time-dependent activity of Pelorgonium sidoides extract on SH-SY5Y neuroblastoma cells was investigated by XTT assay. The change in the oxidative stress marker 8-Hydroxy-2′-deoxyguanosine (8-OhDG) level was determined by ELISA for the doses applied to the control group root extract at a concentration of 25 μg/mL. Total antioxidant status (TAS) and total oxidant status (TOS) were measured from the cells in the study group with the help of a commercial kit. The oxidative stress index (OSI) was calculated by dividing the TAS by the TOS and multiplying by 100. In order to evaluate the expression levels of apoptosis-related Bax, Bcl-2, Caspase-3, Caspase-8, and Caspase-9 genes at the mRNA level in control and dose group cells, RNA isolation was performed from the SH-SY5Y control and dose group cells (IC50 value). *Results:* It is observed that the *P. sidoides* substance inhibits proliferation in cells at 24 h (*p* < 0.05). As the dose increases, cell proliferation decreases (*p* < 0.05). The IC50 value was calculated to be 113.83 μg/mL at 24 h. The concentration of 8-OhDG increased in neuroblastoma cells as a result of *P. sidoides* extract treatment (*p* < 0.05). TOS levels increased in neuroblastoma cells treated with *P. sidoides* extract (*p* < 0.01). OSI levels increased in cells treated with *P. sidoides* extract (*p* < 0.001). BAX and Caspase-8 expression increased are statistically significant in the *P. sidoides* dose group (*p* < 0.05). *Conclusions: P. sidoides* extract induces apoptosis in neuroblastoma cells through oxidative stress and mitochondrial- and death receptor-mediated pathways. This study highlights the potential of *P. sidoides* as a complementary therapeutic agent for neuroblastoma, warranting further in vivo and clinical investigations to assess its safety and efficacy.

## 1. Introduction

Childhood cancers stand out as a serious public health problem in the medical field. Every year, thousands of children are diagnosed with cancer worldwide, and these diseases are responsible for a significant portion of childhood deaths [1]. Early diagnosis and treatment of these cancers are of great importance for both the course of the disease and the quality of life of patients. Cancers seen in childhood generally originate from cells with high proliferative potential, and in addition to genetic factors, environmental effects also play a role in the emergence of these diseases [2].

Among childhood cancers, neuroblastoma holds a particularly critical position. It is the most common extracranial solid tumor diagnosed in children and accounts for approximately 15% of all pediatric cancer-related deaths [3]. Originating from primitive neural crest cells in the embryonic period, this tumor is the third most common type of cancer in childhood, with a rate of 7% [4,5]. Neuroblastoma, which shows great clinical and genetic heterogeneity, presents challenges that limit the effectiveness of current treatment methods [3,6]. The pathogenesis of neuroblastoma involves a complex interplay of genetic, epigenetic, and environmental factors. Approximately 90% of neuroblastoma patients exhibit chromosomal changes, including MYCN amplification, ALK mutations, and 1p or 11q deletions, which are linked to disease progression and poor outcomes [7]. These alterations underscore the need for targeted therapies that can address these genetic aberrations. Current treatment strategies for neuroblastoma typically include surgery, chemotherapy, radiotherapy, and immunotherapy. However, these approaches often come with significant toxicity, particularly in young patients, and are limited in their efficacy for high-risk cases. As such, there is an urgent need for novel therapeutic strategies that can improve survival while minimizing side effects.

In recent years, intensive studies have been conducted on the role of plant-based bioactive molecules in cancer treatment [8,9]. It has been shown that these molecules have the potential to suppress tumor proliferation, activate apoptosis mechanisms, and regulate oxidative stress levels [10,11]. Pelargonium sidoides (*P. sidoides*) root extract has been used in traditional treatment methods for a long time and has been found to be effective in modern medicine, especially in the treatment of upper respiratory tract infections [12,13]. However, recent studies also focus on the potential anticancer effects of *P. sidoides* and show that this plant may affect various biological pathways associated with cancer [14]. The phenolic compounds of *P. sidoides* attract attention with their potential to regulate PI3K-Akt and Ras/MAPK pathways, which are cancer-related signaling pathways [15].

The aim of this study was to investigate the effects of *P. sidoides* root extract on the SH-SY5Y human neuroblastoma cell line. The effects of the extract on cell proliferation, oxidative stress levels and apoptosis mechanisms were examined with various experimental methods, and the findings are presented in a way that will contribute to new treatment approaches for childhood cancers.

## 2. Materials and Methods

### 2.1. Study Design and Study Population

This study was supported by the Ordu University scientific research project (BAP project number: A-2308). The study was conducted between 6 June 2023, and 29 May 2024.

### 2.2. Cell Culture

For the cell culture, SH-SY5Y human neuroblastoma cells were thawed, cultured, and maintained under appropriate conditions for experiments. Cell proliferation was achieved using DMEM-F12 culture medium containing 10% fetal bovine serum, 2 mM L-glutamine, and 1% penicillin–streptomycin in a 95% humidity and 5% CO_2_ oven at 37 °C. Cells were multiplied under appropriate conditions and made ready for experiments. In order to renew our stocks, they were frozen in new cryos with DMSO and stored at minus 80 °C.

### 2.3. XTT Method

The dose- and time-dependent activity of Pelorgonium sidoides extract on SH-SY5Y neuroblastoma cells was investigated by XTT assay. Briefly, cells plated in 96-well plates were treated with the synthesized substance at appropriate dose ranges specified in the literature for 24, 48, and 72 h, following an initial incubation period of 24 h. After incubation, XTT solution was added to the 96-well plates, and after 4 h, absorbance values (OD) were measured using an ELISA reader at a wavelength of 450 nm with a reference range of 630 nm. The percentage of cell viability was calculated by dividing the optical density value measured in each well by the control optical density value and multiplying by one hundred. Then, non-toxic doses were determined, and dose selection was made for further experiments. The inhibitory concentration (IC50) value was calculated via the (https://www.aatbio.com/tools/ic50-calculator, accessed on 20 December 2023). % Cell viability = [(measured optical density value)/(control optical density value)] × 100.

### 2.4. ELISA Tests

The change in the oxidative stress marker 8-Hydroxy-2′-deoxyguanosine level was determined by ELISA for the doses applied to the control group root extract at a concentration of 25 μg/mL. A human 8-Hydroxy-deoxyguanosine ELISA kit (Cat No: EA0048Hu; Bioassay Technology Laboratory, Shanghai, China) was used. The application procedure was as follows: First, the standard concentration provided with the kit was prepared by serial dilution. Standard concentrations were adjusted to be 800, 400, 200, 100, and 50 ng/mL from the main stock of 1600 ng/mL. Then, 20 mL of wash solution provided as 30X was diluted to 1X with distilled water. Kit components were brought to room temperature, and the study began. Standards were loaded into 96-well ELISA plates as 50 μL. No antibody was added to the standards. Samples were loaded as 40 μL and then 10 μL of anti-8-OHDG antibody was added. Then, 50 μL of streptavidin–HRP was added to both standards and samples. This addition was not made for the blank well. After all these added components, incubation was applied for 60 min at 37 °C in the dark. At the end of the incubation, washing was performed 5 times for all wells with wash solution. Then, substrate solution A and 50 μL of substrate solution B were pipetted. Then, incubation was performed for 10 min at 37 °C in the dark. After the incubation was completed, 50 μL of stop solution was added and readings were performed using a microplate reader (Epoch 2) at 450 nm within 10 min. A similar protocol was applied for all other ELISA tests.

### 2.5. Determination of Total Antioxidant Status (TAS), Total Oxidant Status (TOS), and Oxidative Stress Index (OSI)

The principle of total oxidant level measurement is based on the conversion of the ferrous ion chelator complex of the oxidants in the sample to ferric ion, which reacts with the chromogen in an acidic environment and causes an increase in absorbance. The increase in absorbance observed spectrophotometrically is directly proportional to the oxidant molecules in the sample [16,17]. The principle of total antioxidant level measurement is based on the principle that all antioxidants in the sample convert the blue–green ABTS radical into colorless reduced ABTS [18,19]. In this study, a control and dose group were created for SH-SY5Y neuroblastoma cells in order to investigate the effect of *P. sidoides* root extract. Total oxidant and total antioxidant levels were measured from the cells in the study group with the help of the commercial kit provided (TAS and TOS, Rel Assay, Gaziantep, Turkey. OSI was calculated by dividing the TAS by the TOS and multiplying by 100. The experiments were repeated three times to ensure the reliability of the experiments [20].

### 2.6. Total RNA Isolation, Quality Control of RNA Samples, and cDNA Synthesis and RT-PCR

In order to evaluate the expression levels of apoptosis-related Bax, Bcl-2, Caspase-3, Caspase-8, and Caspase-9 genes at the mRNA level in control and dose group cells, RNA isolation was performed from the SH-SY5Y control and dose group cells (IC50 value). Neuroblastoma cells were plated in 6-well cell culture plates at a density of 1 × 10^5^ to 2 × 10^5^ cells per well and incubated for 24 h to achieve appropriate confluency for further experiments. Then, control and dose groups were adjusted. At the end of incubation, the medium in the wells was removed and the cells were washed with 3 mL cold PBS. After removing the PBS, 500 μL Trizol was added to the wells to lift the cells. The homogenate was transferred to 1.7 mL Eppendorf tubes and incubated for 10 min at room temperature. Then, 100 μL of chloroform was added to each Eppendorf tube and vortexed, and incubated again at room temperature for 15 min. Then, the tubes were centrifuged at 15,000× *g* for 15 min at 4 °C, and the colorless upper phase containing RNA was collected and transferred into separate Eppendorf tubes. A total of 250 μL of isopropanol was added to the collected upper phase, pipetted, and left at room temperature for 10 min. After the tubes were centrifuged at 15,000× *g* for 10 min at +40 °C, the supernatant was carefully discarded, and 70% ethanol was added to the pellet and centrifuged at 12,000× *g* for 10 min at +40 °C. The supernatant then was discarded again and the pellet was air dried for a short time. Finally, the pellet was dissolved with 40 μL of RNase–DNase-free water.

The quantity and quality of the obtained RNAs were determined using Nanodrop. A260/A280 and A260/A230 ratios were evaluated to determine the phenol, protein, and genomic DNA contamination that may occur during isolation. After UV measurements, RNA samples with 2 ± 0.1 for A260/A280 and 2.0–2.4 for A260/A230 were used in the analyses.

For mRNA expression analysis, cDNA synthesis was performed from isolated RNAs using the A.B.T.™ cDNA Synthesis Kit with RNase Inhibitor (High-Capacity) synthesis kit (CatNo: C03-01-20, ABT, Ankara, Turkey) along with a random hexamer, dNTP, and reverse transcriptase enzyme (RT) followingg the manufacturer’s protocol. After the mixture was prepared, it was incubated at 25 °C for 10 minutes and then at 37 °C for 120 minutes for cDNA synthesis. At the end of this period, the mixturewas kept at 85 °C for 5 min to inhibit the enzyme. The synthesized cDNAs were stored at −20 °C until RT-PCR.

Expression levels of apoptosis-related genes at the mRNA level in the control and dose group cells were evaluated using a real-time PCR system (Rotor Gene, Qiagen, USA). SYBR Green, which can bind to double-stranded DNA, was used as the dye. The reaction was completed with 5X SYBR Green mixture, 5 pMol forward (F), 5 pMol reverse primer (R), 2 μL cDNA, and sterile RNase–DNase-free water to a final concentration of 1X, and the reaction was carried out in sterile 8-well PCR strips. The temperature profile of the reaction was adjusted to 95 °C for 15 min and then 40 cycles (95 °C for 15 s, 60 °C for 20 s, and 72 °C for 20 s) took place. Then, the heating process was carried out gradually from 60 °C to 95 °C with 0.5 °C increments, and optical measurements were made at each 0.5 °C increment in temperature to create the melting curve graph via melting curve analysis. The data obtained from the optical analysis in real-time PCR were recorded as “Ct” (cycle of threshold), and the expression levels of the target genes were normalized with the GAPDH reference gene. The sequences were obtained from the OriGene (https://www.origene.com, accessed on 1 October 2023) online web page.

### 2.7. Statistical Analysis

All statistical analyses were performed using SPSS software (Statistical Package for the Social Sciences version 21, Chicago, IL, USA). Real-time PCR data were analyzed using the ΔΔCT method and quantification was performed with the help of a computer program. Volcano plot analyses in the web-based “RT^2^ Profiler™ PCR Array Data Analysis” program were used. The method was based on the comparison of two expression results ±3SD. Thus, in cases where gene expression was compared, the expression values of relevant genes in the control and dose groups were determined relatively. The comparison of the groups was evaluated statistically using the “Student *t*-test” analysis in the “RT^2^ Profiler™ PCR Array Data Analysis” program. Continuous variables are given as mean ± standard deviation, and categorical variables are given as a number and percentage. A *p*-value of <0.05 was considered statistically significant.

## 3. Results

### 3.1. Reduced Cell Viability Induced by P. sidoides Extract in SH-SY5Y Neuroblastoma Cells

**Figure 1:**
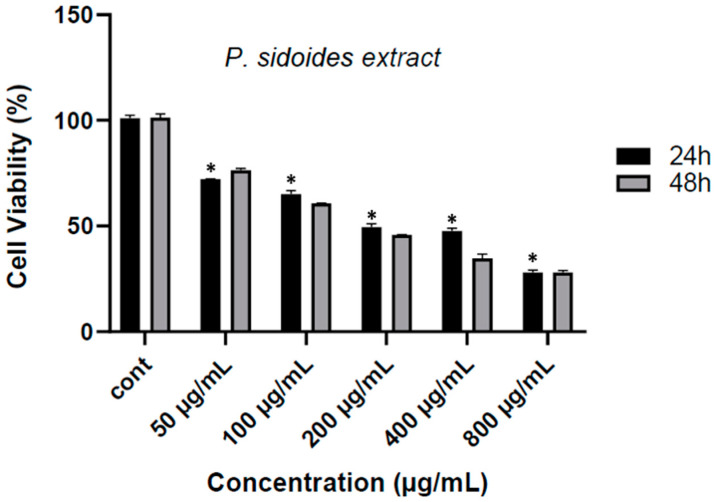
Cell viability at various concentrations in SH SY5Y human neuroblastoma cell lines treated with *P. sidoides* extract at 24 and 48 h. *: *p* < 0.05.

The effects of various concentrations on cell viability in SH-SY5Y human neuroblastoma cell lines to which *P. sidoides* extract was applied at 24 and 48 h are shown in Figure 1. While cell viability in the control group cells was accepted as 100%, cell viability decreased significantly at the end of 24 h at a concentration of 50 µg/mL of the extract to approximately 70% (*p* < 0.05). A gradual decrease in cell viability was observed depending on the increasing concentrations of the extract: Cell viability decreased to 65% at a concentration of 100 µg/mL (*p* < 0.05); viability decreased to 55% at a concentration of 200 µg/mL (*p* < 0.05); viability decreased around 50% at 400 µg/mL; and viability decreased to 40% at a concentration of 800 µg/mL (*p* < 0.05). A similar trend was observed during the 48 h incubation period, and it was determined that the cytotoxic effect of *P. sidoides* continued stably as time progressed. These findings provide support that *P. sidoides* root extract significantly reduced cell viability in a dose- and time-dependent manner, and the IC50 value was 113.83 µg/mL (Figure 1).

### 3.2. Increased Oxidative DNA Damage Due to P. sidoides Extract Treatment

The IC50 value was calculated to be 113.83 μg/mL at 24 h. The IC50 (113.83 μg/mL) value determined as the dose group was used in the following experimental steps. The protein concentrations of the control group SH-SY5Y cells and the *P. sidoides* extract dose group (113.83 μg/mL) cells were compared with the 8-Hydroxy-deoxyguanosine ELISA test. 

**Figure 2:**
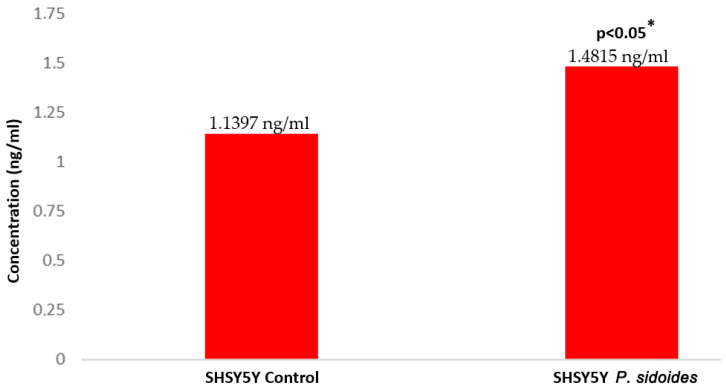
Comparison of 8-Hydroxy-deoxyguanosine (8-OhDG) concentration in *P. sidoides* extract-applied dose group cells compared to control. *: Student’s *t*-test.

The concentration calculation was analyzed according to Figure 2 and this value was determined as 1.1397 ng/mL in the control group, while this value was determined as 1.4815 ng/mL in the extract dose group (*p* < 0.05) (Figure 2). These results show us that the concentration of 8-Hydroxy-deoxyguanosine, one of the important oxidative stress and genotoxicity markers, increased in neuroblastoma cells as a result of *P. sidoides* extract treatment (*p* < 0.05). This result indicates that *P. sidoides* extract may have increased the possible oxidative stress and genotoxic effect in the cell and led to cell death.

### 3.3. Oxidative Stress Induced by P. sidoides Extract Alters TAS, TOS, and OSI Levels

**Figure 3:**
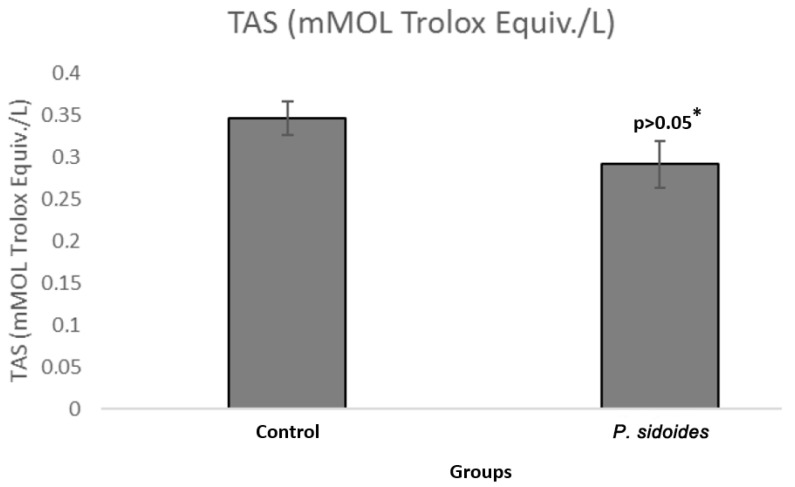
Comparison of total antioxidant status (TAS) levels in the control group and *P. sidoides* extract-treated cells. *: Student’s *t*-test.

When the control and *P. sidoides* group cells were compared, the TAS value was 0.3455 mMOL Trolox Equiv./L for the control group, while it was 0.2915 mMOL Trolox Equiv./L in the *P. sidoides* dose group cells. This result shows that the total antioxidant activity in SH-SY5Y cells treated with *P. sidoides* extract was partially reduced (*p* > 0.05) (Figure 3).

**Figure 4:**
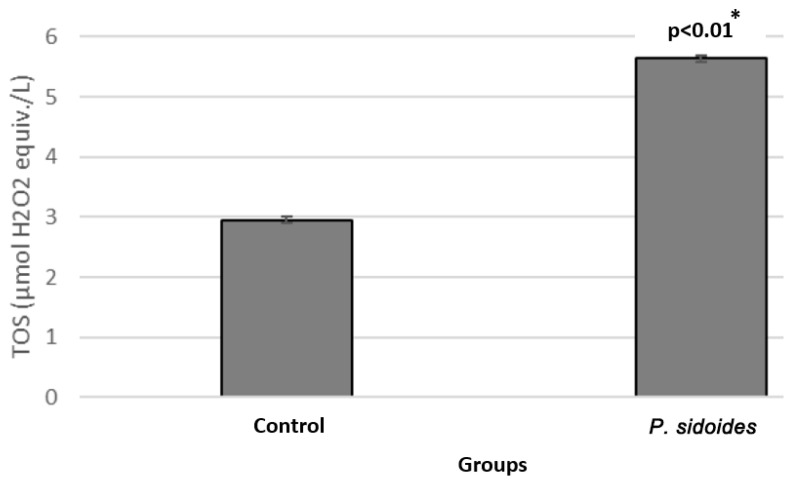
Comparison of total oxidant status (TOS) levels in the control group and *P. sidoides* extract-treated cells. *: Student’s *t*-test.

When the control and *P. sidoides* group cells were compared, the TOS value was determined as 2.9463 µmol H_2_O_2_ equiv./L for the control group, while it was 5.6370 µmol H_2_O_2_ equiv./L in the *P. sidoides* group cells. This result shows that the total oxidant activity increased in neuroblastoma cells treated with *P. sidoides* extract (*p* < 0.01) (Figure 4).

**Figure 5:**
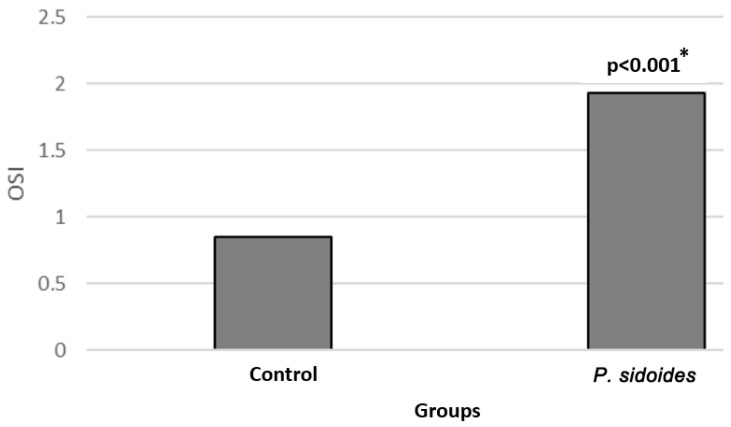
Comparison of OSI (oxidative stress index) values in control group and *P. sidoides* extract-treated cells. *: Student’s *t*-test.

The OSI values are shown in Figure 5. OSI levels increased in cells treated with *P. sidoides* extract (*p* < 0.001). While the OSI value was determined to be approximately 0.9 in the control group, this value increased to 1.8 in the *P. sidoides* group (Figure 5).

### 3.4. Activation of Apoptotic Pathways in SH-SY5Y Cells by P. sidoides Extract

The gene expression fold changes in the *P. sidoides* dose group and control group SH-SY5Y cells are shown in Table 1. BAX gene expression, caspase-3 expression, caspase-8 expression, and caspase-9 gene expression increased 2.93, 3.31, 4.34, and 2.41-fold in the *P. sidoides* dose group compared to the control at the mRNA level, respectively. Among these changes, the BAX and Caspase-8 expression increases were statistically significant in the *P. sidoides* dose group (*p* < 0.05). There was no significant change in Bcl-2 gene expression (Table 1).

## 4. Discussion

This study comprehensively examined the effects of *P. sidoides* root extract on human neuroblastoma cell line (SH-SY5Y). The findings show that *P. sidoides* extract can inhibit cell proliferation and increase oxidative stress and genotoxic effects, leading to cell death.

In our study, the XTT test results revealed that *P. sidoides* significantly reduced cell proliferation at the dose where it reached the IC50 value. This supports the anticancer potential of the extract. *P. sidoides* is rich in phenolic compounds, which suggests that it activates mechanisms that suppress cell proliferation. Studies in the literature show that phenolic compounds generally play a role as cell cycle regulators and apoptosis inducers [21,22]. Ramirez et al. reported that plant polyphenols can stop the growth of cancer cells by inhibiting the PI3K/Akt and Ras/MAPK signaling pathways [21]. *P. sidoides* is also thought to inhibit cell proliferation in the neuroblastoma cell line through similar mechanisms.

In our study, measurements performed with ELISA revealed that *P. sidoides* affects DNA damage and is an important indicator of oxidative stress by increasing 8-Hydroxy-deoxyguanosine levels. These findings suggest that cell death is induced through oxidative stress and genotoxicity mechanisms. It has been stated in the literature that *P. sidoides* may cause genotoxic effects by increasing oxidative stress levels [15,23,24,25]. Pereira et al. showed that the phenolic components of *P. sidoides* extract triggered apoptosis in cancer cells by regulating oxidative stress and increasing reactive oxygen species (ROS) levels [15,23]. Similarly, Mates et al. reported that increased oxidative stress leads to DNA damage, which in turn induces apoptosis in cancer cells [24]. Our study supports these literature findings and shows that *P. sidoides* may be an effective herbal agent, especially in increasing oxidative stress.

In our study, TAS, TOS and OSI results show that *P. sidoides* increases total oxidant levels while decreasing antioxidant levels and significantly increases oxidative stress index (OSI). These results support the critical role of oxidative stress in inducing apoptosis in neuroblastoma cells. Increased oxidative stress leads to the accumulation of reactive oxygen species (ROS), causing DNA damage, mitochondrial dysfunction and disruptions in cellular homeostasis [25,26,27,28]. Yuhang et al. stated that ROS accumulation causes loss of mitochondrial membrane potential and plays a key role in the initiation of apoptosis [28]. Consistent with these findings, our study shows that *P. sidoides* triggers apoptosis in neuroblastoma cells by increasing oxidative stress.

In our study, the RT-PCR results revealed significant increases in BAX, caspase-3, caspase-8, and caspase-9 gene expression. This suggests that both the mitochondrial pathway (BAX) and death receptor-mediated pathways (Caspase-8) are activated. In particular, high regulation of BAX and caspase-8 leads to the opening of mitochondrial permeability transition pores, supporting cytochrome C release and the subsequent activation of the apoptotic cascade. These findings are consistent with the report by Chaudhry et al. that herbal compounds induce apoptosis by activating the BAX and caspase pathways [29]. In addition, the increased expression of caspase-3 and caspase-9 indicates that apoptosis progresses via the mitochondrial pathway [30]. It has been previously reported in the literature that phenolic compounds of *P. sidoides* promote apoptosis by regulating the expression of these genes [15,31]. Young et al. reported that phenolic compounds contribute to the direction of programmed cell death in cancer cells by increasing caspase-3 and caspase-9 activity [31]. Our study shows that these mechanisms can also be activated by *P. sidoides* in the neuroblastoma cell line. In addition, no significant change was observed in Bcl-2 gene expression. This suggests that anti-apoptotic mechanisms are suppressed and that pro-apoptotic signals become dominant during the apoptosis process. It has been frequently stated in the literature that the decrease in the Bcl-2/BAX ratio is a critical indicator for apoptosis [32]. The observation that this ratio was disrupted in our study is strong evidence of the apoptosis-promoting effect of *P. sidoides*.

The IC50 value obtained in our study (113.83 µg/mL) is consistent with previous studies. For example, Pereira et al. showed the antiproliferative effect of *P. sidoides* on a leukemia cell line at similar dose ranges [15]. Similarly, it has been reported that doses in the range of 100–400 µg/mL are generally used in studies for the effects of plant extracts on cancer cells [23,29]. This study is one of the first studies to confirm the apoptotic effect of *P. sidoides* on a neuroblastoma cell line.

### Limitations of the Study

In our study, only one cancer cell line (SH-SY5Y) was used. However, evaluating the potential cytotoxic effects of plant extracts on healthy cells is critical for treatment efficacy and safety. In future studies, the selectivity of *P. sidoides* should be investigated using healthy cell lines (e.g., fibroblast or epithelial cells). It is known that non-selective agents are not suitable for clinical application. In this study, only the neuroblastoma cell line was examined, and no reference cell line was used. Comparative analyses with healthy cell lines and different cancer cell lines (such as HeLa or MCF-7) are needed to determine whether apoptosis is specifically induced in cancer cells. Such analyses will more clearly reveal the targetability and therapeutic potential of *P. sidoides*. While this study demonstrates significant fold changes in gene expression for several apoptosis-related genes in SHSY5Y cells using RT-PCR, one of the limitations is the absence of protein-level validation through Western blot analysis. Western blotting could provide additional confirmation of changes in protein levels for key genes, such as BAX, Caspase-3, and Caspase-8, as supported by previous studies [29,31]. Future studies should include Western blot analysis to validate these findings at the translational level and further substantiate the apoptotic effects of *P. sidoides* extract.

## 5. Conclusions

This study investigated the effects of Pelargonium sidoides root extract on human neuroblastoma cell line (SH-SY5Y) to reveal the anticancer potential of this herbal agent. The results obtained show that *P. sidoides* can induce apoptosis by inhibiting cell proliferation and increasing oxidative stress and genotoxicity. It was determined that phenolic compounds in particular promote cancer cell death by activating oxidative stress mechanisms and regulating apoptotic pathways. Our results indicate that *P. sidoides*, rich in phenolic compounds, can be evaluated as a complementary agent in the treatment of pediatric cancers, such as neuroblastoma. However, further in vivo and clinical studies are needed to evaluate its clinical applicability and safety. In addition, the potential of this herbal agent should be investigated in more detail with different doses, application times, and combined treatment strategies.

## Figures and Tables

**Table 1:** Gene expression fold changes in the *P. sidoides* dose group and control group SH-SY5Y cells. The RT^2^ Profiler™ PCR Array Data Analysis programs were used.

Gene	Fold Change	*p*-Value
GAPDH	1.00	-
BAX	2.93	0.017
BCL2	−1.03	0.975
CASPASE-3	3.31	0.102
CASPASE-8	4.34	0.024
CASPASE-9	2.41	0.093

## Data Availability

Data is available upon request to the corresponding author.
